# Failure Behavior and Constitutive Model of Weakly Consolidated Soft Rock

**DOI:** 10.1155/2013/758750

**Published:** 2013-12-29

**Authors:** Wei-ming Wang, Zeng-hui Zhao, Yong-ji Wang, Xin Gao

**Affiliations:** ^1^College of Civil Engineering of Shandong University of Science and Technology, Qingdao, Shandong 266590, China; ^2^State Key Laboratory of Mining Disaster Prevention and Control Cofounded by Shandong Province and the Ministry of Science and Technology, Qingdao, Shandong 266590, China

## Abstract

Mining areas in western China are mainly located in soft rock strata with poor bearing capacity. In order to make the deformation failure mechanism and strength behavior of weakly consolidated soft mudstone and coal rock hosted in Ili No. 4 mine of Xinjiang area clear, some uniaxial and triaxial compression tests were carried out according to the samples of rocks gathered in the studied area, respectively. Meanwhile, a damage constitutive model which considered the initial damage was established by introducing a damage variable and a correction coefficient. A linearization process method was introduced according to the characteristics of the fitting curve and experimental data. The results showed that samples under different moisture contents and confining pressures presented completely different failure mechanism. The given model could accurately describe the elastic and plastic yield characteristics as well as the strain softening behavior of collected samples at postpeak stage. Moreover, the model could precisely reflect the relationship between the elastic modulus and confining pressure at prepeak stage.

## 1. Introduction

Coal seams in China's western area of Xinjiang, Inner Mongolia, and Ningxia are mainly located in measure strata composed of Jurassic and cretaceous soft rock which show poor characteristics of low strength, easy to be weathered, and easy to collapse. These weak mechanical properties bring difficulties to the construction and stability maintenance of rock tunnel. After excavation of roadway, some mine disasters such as large range of roof fall, terrible floor heave, sudden coal bump, and landslides may be induced which bring a serious threat to the production safety and economic benefits. Therefore, it is urgent to make clear the failure characteristics and constitutive relations of typical soft rock and coal rock in these engineering sites. This subject is of very important engineering value for the prevention of mining dynamical disasters and stability maintenance of roadway.

Some research achievements about mechanical behaviors of coal and rock sample have been reported by domestic and foreign scholars. Zuo et al. [1] carried out indoor tests by uniaxial and triaxial compression for sandstone, respectively, and came to the conclusion that the type II curve during rock damage could be obtained by control of circumferential displacement. Lu et al. [2] and Zhang et al. [3] studied the attenuation rules of mechanical parameters of rock mass at postpeak stage by triaxial compression test. Based on the laboratory test results of silk screen chlorite schist, Li et al. [4] established its mechanical model which considered the behavior of dilatancy, stain hardening and softening. The energy dissipation feature of mudstone at failure was analyzed through triaxial compression test by Han et al. [5] which showed that the actual dissipation energy of mudstone at failure was enhanced with the increasing of confining pressure. In addition, Chong et al. [6] studied the influences of blasting disturbance, water content, and sample bedding on the strength behavior of sandstone by uniaxial and triaxial compression tests. According to the research findings of constitutive model, He and Kong [7], Zhang et al. [8], and Jiang et al. [9] established the constitutive relation of rock and soil body, respectively, by employing the improved nonlinear elastic model of Duncan-Zhang. Huang and Subhash [10], Paliwal and Ramesh [11], and Graham-Brady [12] put forward some stress-strain relations for rock mass under uniaxial compression through the theory of fracture mechanics. Based on the Weibull probability distribution theory of microunit strength and taking the probabilistic damage variable, Cao et al. [13], Liu et al. [14], Li et al. [15], and Yang and Wang [16] set up several isotropy statistical damage constitutive models for rock mass which considered the random distribution of inner defects in rock mass. Actually, the strain softening behavior of rock mass at postpeak stage is the result of strain localization which is manifested as non-uniform deformation at failure stage. Based on this point, Fan et al. [17], Wang [18], and Chen and Pan [19] presented some different constitutive models considering strain localization based on work-energy principle.

Results of comparison between numerous indoor experiments show that rock mass present different mechanical behaviors in different regions because of the complexity of geological conditions and the distribution discreteness of rock mass. Even in the same area, obvious differences also exist in rock samples which were taken from different positions. So the above research results and models are not suitable for the soft rock in the western mining area. In the current research on constitutive model, statistical damage model which preferably associates the extension of micro-crack with the degradation of mechanical parameters can give out accurate results for the description of mechanical behavior of rock mass. However, the present researches did not consider the influence of confining pressure on elastic modulus at the prepeak stage as well as the effect of stress state changes on constitutive model. In view of this, the failure mechanisms and constitutive models of typical weakly consolidated mudstone and coal samples in western mining area under different stress states were studied in this paper. The conclusions obtained may provide theoretical basis for further numerical calculation and engineering application.

## 2. Sample Preparation and Test Method

**Figure 1:**
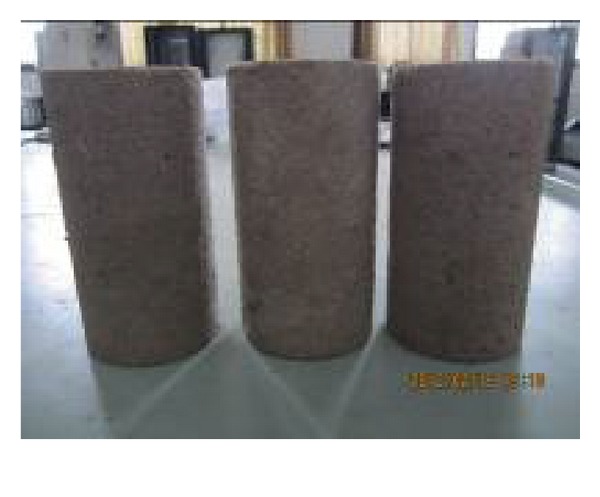
Mudstone samples for triaxial compression test.

The test samples of mudstone and coal rock were all collected from level transportation entry of NO. 4 mine of Xinjiang Ili area whose formation belongs to the typical weakly consolidated soft rock. In order to maintain the natural moisture content, the specimens were packaged immediately after sampling. With the poor characteristics of weak cementation and low strength, transfixion cracks often appear during the preparation of mudstone samples, and success rate is only about 35%. According to the national standard, all samples were processed into cylinder with a diameter of 50 mm, and 100 mm high. The group numbers of mudstone and coal samples is all 3 to 5 for uniaxial and triaxial compression tests, respectively, as shown in Figure 1. The sample number was recorded as A-B-i where A stands for compression style (among them D denoted uniaxial compression, and S was triaxial compression), B represented sample type (among them N denoted mudstone, and M stands for coal rock), and i was sample number.

The type of servo triaxial testing machine is TAW-2000. Firstly, a preload of 0.2 KN was applied on the sample to ensure the close contact between rock specimen and loading device. Then, axial compression displacement load at rate of 0.2 mm/min was applied in both uniaxial and triaxial compression tests until the samples were destroyed. During the triaxial compression test, confining pressure was increased to the predetermined value (in triaxial compression tests, the confining pressure was set as 1 MPa, 3 MPa, and 5 MPa, resp.) first at rate of 15 N/S and then maintained constant. Its variation scope was less than 2% of the predetermined value.

## 3. Failure Behavior of Weakly Consolidated Soft Rock under Uniaxial Compression

### 3.1. Mechanical Behavior of Mudstone

**Figure 2:**
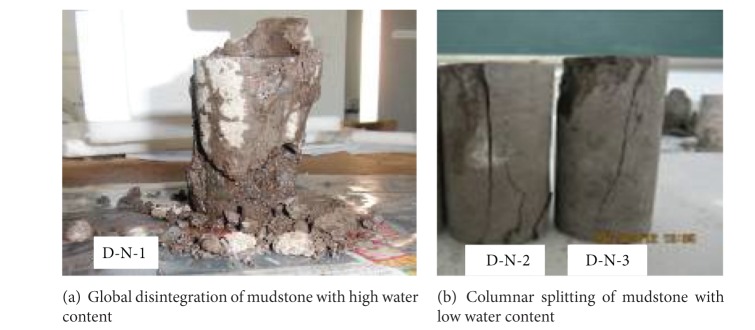
Failure modes of mudstone samples under uniaxial compression with different moisture contents.

There were two typical failure modes for mudstone with different water contents under uniaxial compression. As shown in Figure 2(a), the sample numbered D-N-1 whose water content (16.94%) was close to saturation state suffered global disintegration failure. When the load was added to elastic limit, the sample became crude and short, and some local surface showed scale flaking. For the relatively dry samples with low water content as shown in Figure 2(b) which was numbered as D-N-2 and D-N-3, the main failure style was columnar splitting.

**Figure 3:**
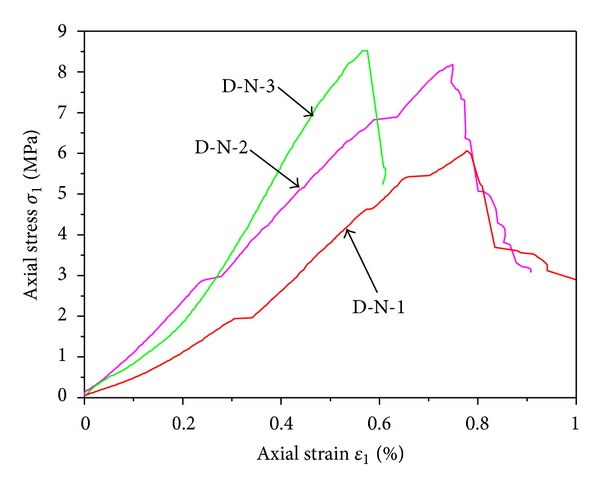
Stress-strain relations of mudstone samples under uniaxial compression with different moisture contents.

Figure 3 showed the stress-strain relation of mudstone samples under uniaxial compression. Obviously, the three typical samples were all damaged rapidly at postpeak stage and almost had no residual strength in the process of loading. The uniaxial compression strength of the three rock specimens were 5.79 MPa, 8.14 MPa, and 8.45 MPa, respectively. So, the strength of mudstone had great dispersion, and the fundamental cause lied in the weak cementation, different water content, preexisting fractures, and the different joint distribution state and density in different samples.

### 3.2. Failure Behavior of Coal Rock under Uniaxial Compression

**Figure 4:**
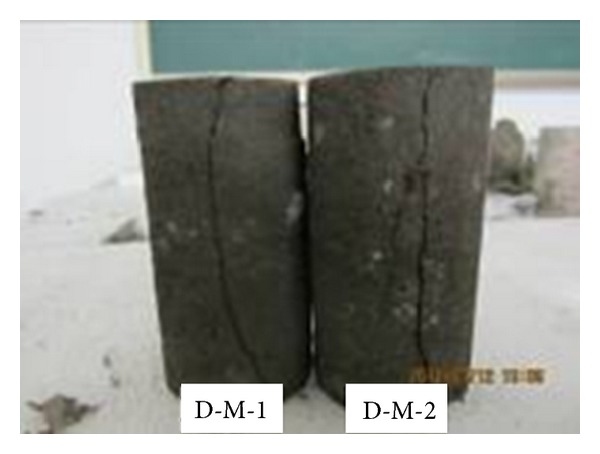
Splitting failure modes of coal samples under uniaxial compression.

The failure modes of coal body under uniaxial compression were illustrated in Figure 4. Overall, columnar splitting was the main failure mode for coal samples in which only one primary damage crack appeared and no local breakage phenomenon presented. The sample mainly suffered tensile damage under axial load and kept relatively intact after failure. The result showed that the pre-existing fractures in coal body were undeveloped.

**Figure 5:**
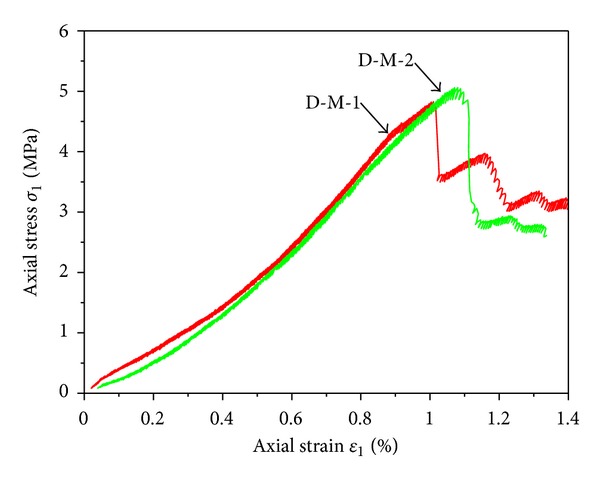
Stress-strain relations of coal samples under uniaxial compression.

From the stress-strain relations (as shown in Figure 5), the curves of two coal samples were basically in coincidence and bear the same variation tendency. The two kinds of coal samples both suffered splitting failure at postpeak stage and no residual strength was retained. However, the peak strength of the samples was quite different from each other where D-M-1 was 4.98 MPa, and D-M-2 was 5.23 MPa. Though there was difference, the difference was not evident. Obviously, the strength of coal rock was lower than that of mudstone. According to Figures 4 and 5, the mechanical properties of coal body in this area showed small discreteness and relative integrity.

## 4. Mechanical Behavior of Soft Rock under Triaxial Compression

### 4.1. Failure Behavior of Mudstone

**Figure 6:**
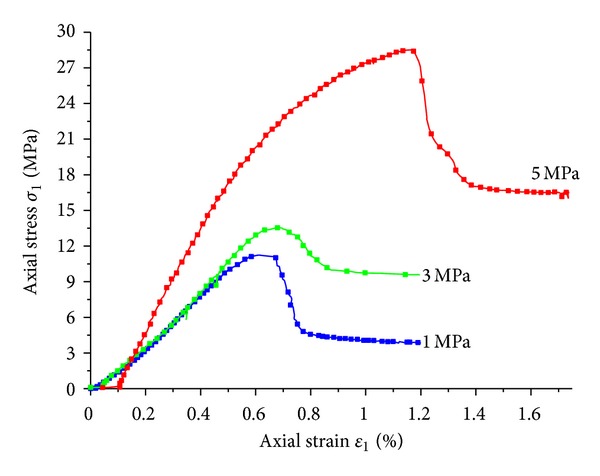
Stress-strain relations of mudstone samples under triaxial compression.

The stress-strain relations of mudstone under confining pressures of 1 MPa, 3 MPa, and 5 MPa were shown in Figure 6. It was obvious that the confining pressure had a significant effect on the shape and deformation feature of the curve. The peak strength and residual strength were both significantly enhanced with the increasing of confining pressure. Similar to hard rock, the complete stress-strain curve of weakly consolidated mudstone could be subdivided into five stages: initial plastic deformation stage, elastic deformation stage, strain hardening stage, strain softening stage, and residual deformation stage. However, the ultimate strain in each stage was much larger than that of normal rock.

**Figure 7:**
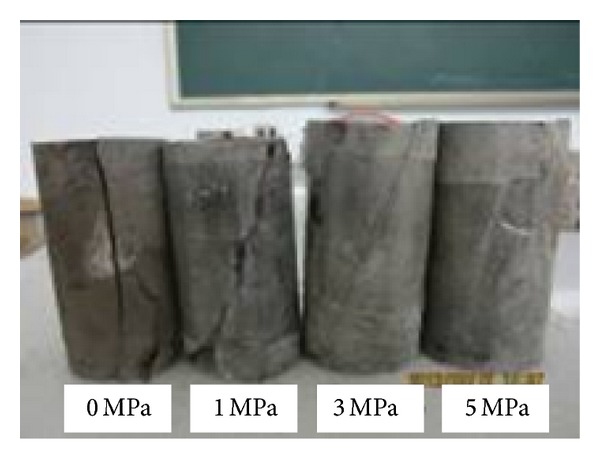
Comparison of failure modes of mudstone samples under different confining pressures.

Because development of internal microcracks in mudstone samples was inhibited by the effect of confining pressure, the failure modes of mudstone were changed from splitting failure to shear failure under triaxial compression. The different failure modes of mudstone samples under confining pressures of 0 MPa, 1 MPa, 3 MPa, and 5 MPa, respectively, were presented in Figure 7. When the confining pressure was set to 1 MPa and 3 MPa, the sample showed simple shear failure and the width of shear band was reduced with the increasing of confining pressure which was completely different from the columnar splitting under uniaxial compression. When the confining pressure was increased to 5 MPa, no obvious failure band appeared on the sample surface, and the failure pattern of mudstone was changed to global plastic softening damage from shear failure. It was thus clear that the increasing of confining pressure effectively inhabited the propagation of primary fractures and shear failure in rock samples caused by the slipping of internal grain.

### 4.2. Damage Characteristics of Coal Rock in Soft Rock Strata

**Figure 8:**
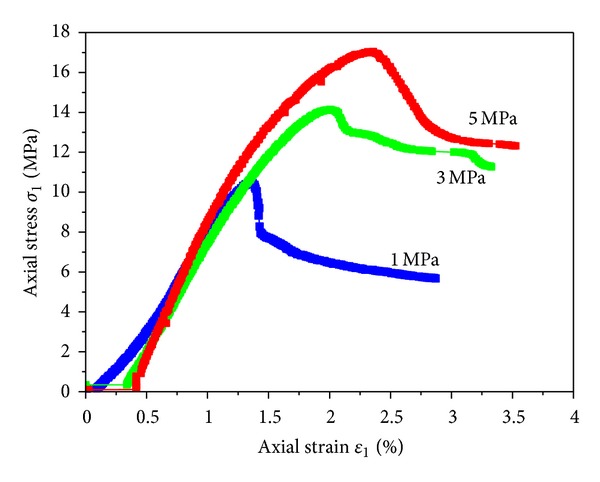
Stress-strain relations of coal samples under triaxial compression.

Figure 8 presented the stress-strain relations of coal samples under triaxial compression. With the increasing of confining pressure, the peak strength and residual strength of coal rock were significantly reinforced, but the elastic modulus at prepeak stage was changed little. The elastic stages at prepeak stage under different confining pressures were basically in coincidence while the peak strengths were moved backward. Large stress drop was shown under low confining pressure after peak point.

**Figure 9:**
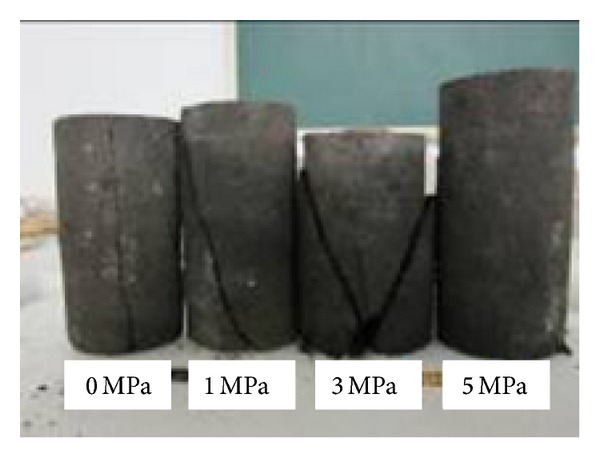
Changes of failure modes of coal samples under different confining pressure.

Comparison of failure modes of coal samples under different confining pressures was shown in Figure 9. The failure mode of coal sample was changed from brittle fracture to plastic shear failure with the increasing of confining pressure. When the confining pressure was set as 1 MPa, it was found that only a single shear band presented which is coincide with the diagonal line of sample and at a 63.4 degree angle to the horizontal line. If the confining pressure was increased to 3 MPa, two conjugate shear zones with the angle of 59.9° to the horizontal line appeared after the sample damage. When the confining pressure continued to be increased to 5 MPa, no obvious shear band appeared and the sample was suffered global plastic softening failure.

**Figure 10:**
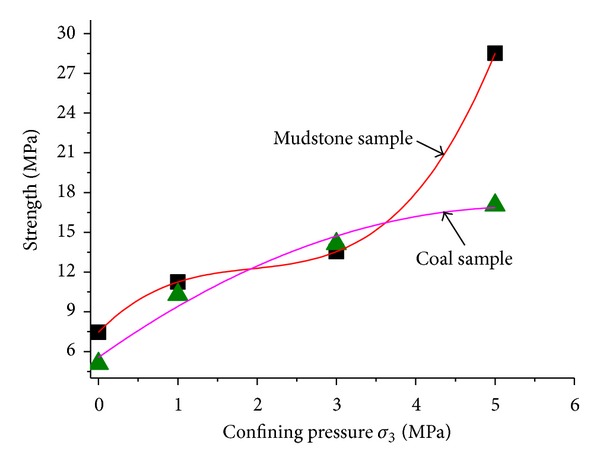
Changes of strengths of mudstone and coal with different confining pressures.

The variation laws of peak strength (mean value of different samples) with different confining pressures for mudstone and coal rock were illustrated in Figure 10. The regression equation for the mudstone strength was

(1) $$\begin{array}{c} \sigma_{c}^{m}=7.46+6.15\sigma_{3}-2.86\sigma_{3}^{2}+0.49\sigma_{3}^{3}. \end{array}$$

The regression equation for the strength was as follows:

(2) $$\begin{array}{c} \sigma_{c}^{m}=5.56+4.24\sigma_{3}-0.39\sigma_{3}^{2}. \end{array}$$

Obviously, the strength of mudstone is more sensitive to confining pressure than that of coal sample. This is the result of the developed primary fractures in mudstone. When the confining pressure is greater than 3 MPa, the strength of mudstone is significant improved with the increasing of confining pressure. By contrast, the strength variation rate of coal rock is relatively slow.

## 5. Constitutive Model of Weakly Consolidated Soft Rock

In order to accurately establish the relationship between mechanical behavior and internal damage, the constitutive model of soft rock was built on the basis of continuum mechanics below. A probabilistic damage factor was defined as follows [20]:

(3) $$\begin{array}{c} D=1-\exp\left(-a\varepsilon^{b}\right), \end{array}$$

where *a* and *b* are fitting parameters. Considering the initial damage and residual strength of rock sample after damage, the damage factor should satisfy: 0 < *D* < 1. Thus, a modification factor *β* was introduced into (3) which was changed as

(4) $$\begin{array}{c} D^{\prime }=1-\beta \exp\left(-a\varepsilon^{b}\right)\;\left(0<\beta <1\right). \end{array}$$

The following relation was deduced according to the Lemaitre hypothesis of equivalent strain [21]:

(5) $$\begin{array}{c} \varepsilon_{1}=\varepsilon_{1}^{•}=\frac{1}{E}\left(\sigma_{1}^{*}-\nu \left(\sigma_{2}^{*}+\sigma_{3}^{*}\right)\right), \end{array}$$

where *σ*_1_*, *σ*_2_*, *σ*_3_* stand for the effective stress, respectively, and

(6) $$\begin{array}{c} \sigma_{1}^{*}=\frac{\sigma_{1}}{1-D^{\prime }},\;\;\sigma_{2}^{*}=\frac{\sigma_{2}}{1-D^{\prime }},\;\;\sigma_{3}^{*}=\frac{\sigma_{3}}{1-D^{\prime }}. \end{array}$$

The constitutive model of weakly consolidated soft rock could be established by the comprehensive utilization of (4) to (6):

(7) $$\begin{array}{c} \sigma_{1}=E\varepsilon_{1}\beta \exp\left(-a\varepsilon^{b}\right)+\nu \left(\sigma_{2}+\sigma_{3}\right). \end{array}$$

**Figure 11:**
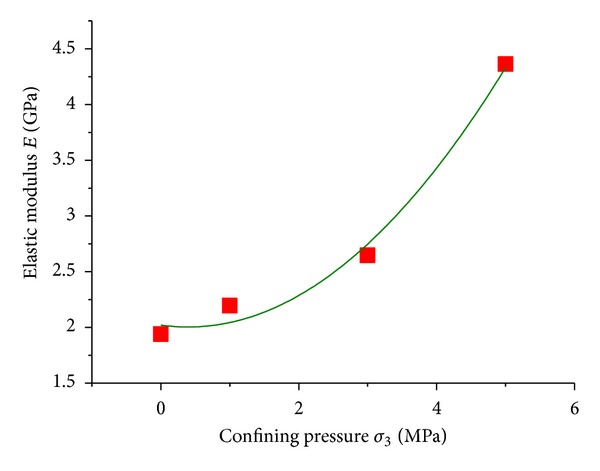
Relation of elastic modulus of mudstone with confining pressure.

Figure 6 indicated that the elastic modulus at prepeak stage of mudstone was concerned with the confining pressure. In order to determine the relation, a scatter diagram to describe the corresponding relation between elastic modulus *E* and confining pressure *σ*_3_ was drawn in Figure 11 where *x*-coordinate denoted *σ*_3_ and *y*-coordinate represented elastic modulus. The elastic modulus was obtained from the average slope of elastic stage in complete stress-strain curve.

Based on the parabolic equation, the data was fitted as

(8) $$\begin{array}{c} E\left(\sigma_{3}\right)=2.02-8.733\sigma_{3}+1.099\sigma_{3}^{2}. \end{array}$$

Therefore, the elastic modulus *E* in (7) should be modified by the above *E*(*σ*_3_). For the conventional triaxial compression test, the principle stress should meet the following relation: *σ*_2_ = *σ*_3_. After taking logarithm twice for both sides of (7), it was changed as

(9) $$\begin{array}{c} {\overset{-}{\sigma }}_{1}=A+b{\overset{-}{\varepsilon }}_{1}, \end{array}$$

in which the equivalent stress was defined as ${\overset{-}{\sigma }}_{1}=\ln\left[-\ln\left(\left(\sigma_{1}-2\nu \sigma_{3}\right)/\alpha E\left(\sigma_{3}\right)\varepsilon_{1}\beta \right)\right]$, the equivalent strain was defined as ${\overset{-}{\varepsilon }}_{1}=\ln\varepsilon_{1}$, and *A* = −*b*ln⁡*a*.

**Figure 12:**
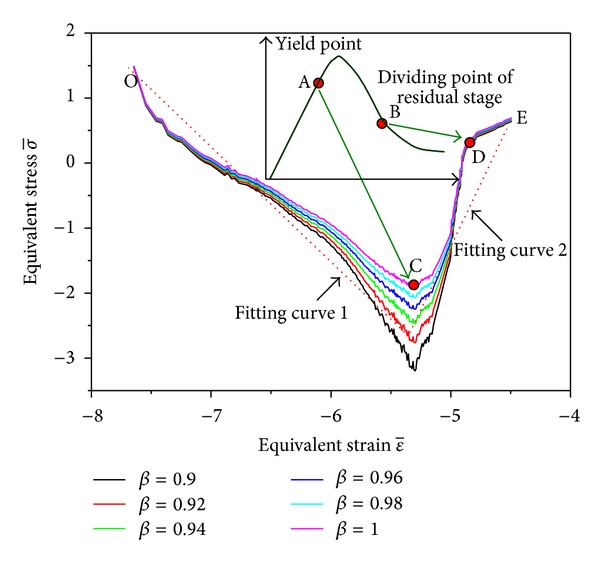
Relation between equivalent stress and equivalent strain when confining pressure was set to 3 MPa.

Evidently, (9) was a linear equation and its fitting parameters could be solved through the series of data points (*σ*_1_, *ε*_1_) under different confining pressures. In order to determine the relationship between equivalent stress and equivalent strain, the data distributions of ${\overset{-}{\sigma }}_{1}$ and ${\overset{-}{\varepsilon }}_{1}$ were calculated as shown in Figure 12 where the range of *β* must satisfies *β* ∈ [0.9,1], and the confining pressure was set to 3 MPa. From the results, ${\overset{-}{\sigma }}_{1}-{\overset{-}{\varepsilon }}_{1}$ curves under different *β* had the same variation trend, but the relations between the two were not simply in linearity. According to the analysis results of experimental data, the turning point C in ${\overset{-}{\sigma }}_{1}-{\overset{-}{\varepsilon }}_{1}$ curve just corresponds to the yield point A at prepeak stage in stress-strain curve, and the turning point D just corresponds to the demarcation point B which lied between upper convex and lower convex at postpeak stage of stress-strain curve. To simplify the calculation, double linear fitting was carried out as shown in Figure 12 where OC and CE stand for the two fitting lines, respectively. Therefore, (9) could be changed as

(10) $$\begin{array}{c} {\overset{-}{\sigma }}_{1}=A_{1}+b_{1}{\overset{-}{\varepsilon }}_{1}\;\varepsilon_{1}\leq \varepsilon_{p},\\ {\overset{-}{\sigma }}_{1}=A_{2}+b_{2}{\overset{-}{\varepsilon }}_{1}\;\varepsilon_{1}>\varepsilon_{p}, \end{array}$$

where *ε*_*p*_ was the axial strain at yield point, and *A*_1_, *b*_1_, *A*_2_, *b*_2_ were the fitting parameters for the two straight fitting lines.

**Figure 13:**
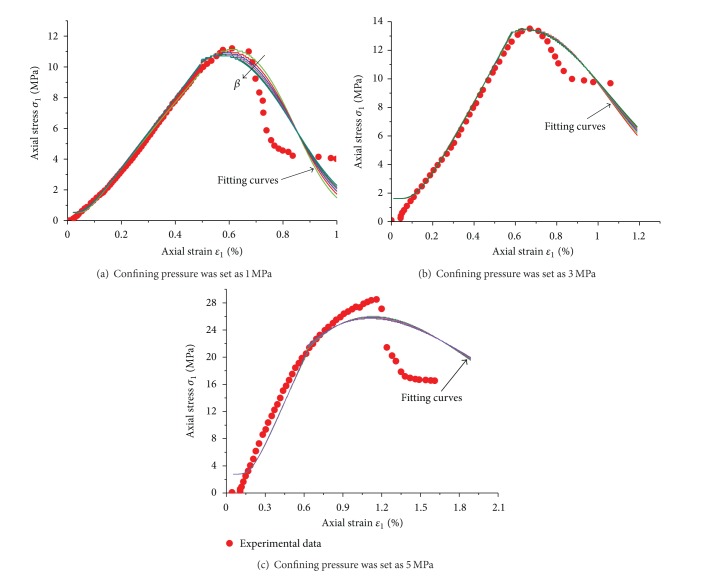
Comparison of fitting curves with experimental data under different confining pressures.

Figure 13 presented the fitting results according to the experimental data under different confining pressures and *β* (the values were set as the same in Figure 12). Obviously, the model could accurately reflect the elastic stage at prepeak stage, the plastic yield feature, and the strain softening behavior of weakly consolidated rock. The confining pressure played an important role to affect the strength and stress-strain curve shape of rock mass. When the confining pressure was set to 1 MPa, value of *β* could determine the peak height of the fitted curve. The peak heights were in good agreement with the test curve when *β* = 0.9. If the confining pressure was increased to 3 MPa and 5 MPa, the fitting curves were basically in coincidence and *β* with different values has no effect on the fitting accuracy. This implied that the initial damage had little effect on the ultimate destruction of rock mass, because these fractures were closed under high confining pressure. This was consistent with the failure pattern in Figure 7 under high confining pressures.

By comparison of fitting curves with experimental data, it is found that the deficiency of this model was all lower convex at postpeak stage which failed to reflect the residual stage with approximate level linear style in Figure 12. The reason was that the experimental data at CE stage was fitted only by one line which would cause error. This problem could be solved by dividing this stage into two parts: CD and DE and then fit them separately. Because of the limited paper space, this work will be dealt with in other separate paper.

**Figure 14:**
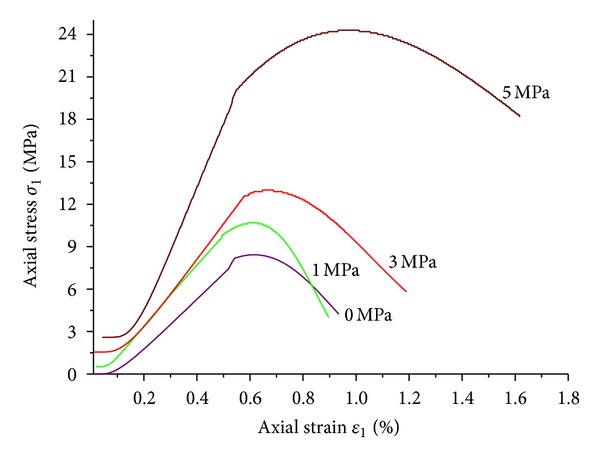
Fitting results of mudstone sample when *β* = 0.9.

Figure 14 presented the fitting results of experimental data under different confining pressures with *β* = 0.9. Compared with Figure 6, the model could accurately reflect the relationship between elastic modulus and confining pressure at prepeak stage. That is the stiffness of test sample would be improved with the increasing of confining pressure.

## 6. Conclusions

(1) The weakly consolidated soft mudstone showed two failure modes under uniaxial compression. One is horizontal expansion and disintegration with high water content; the other is columnar splitting failure with low water content. Under triaxial compression with low confining pressure, coal and rock samples are all presented shear failures. However, if the confining pressure is increased to 3 MPa, no obvious shear dilatancy failure surface appears and the samples suffered global plastic softening failure.

(2) Similar to hard rock, the complete stress-strain curve of soft rock can be divided into the following five stages: initial plastic stage, elastic stage, strain hardening stage, strain softening stage, and residual stage. Nevertheless, the corresponding ultimate strain in each stage is much larger than that of normal rock.

(3) The constitutive model which considers the initial damage of rock sample can accurately describe the behavior of elasticity, plastic yielding, and strain softening, as well as the effect of initial damage on stress-strain curve. Furthermore, the modified elastic modulus can accurately describe the relationship between elastic modulus and confining pressure at prepeak stage. The flaw of this model is that the deformation feature in residual phase cannot be fully reflected.
